# In vitro toxicity of piperazine derivatives involves mitochondrial dysfunction and microtubule-related changes in neuronal cell models

**DOI:** 10.1186/s40360-026-01183-3

**Published:** 2026-07-15

**Authors:** Dina Rönnberg, Stig O.P. Jacobsson

**Affiliations:** Department of Medical and Translational Biology, Umeå University, Umeå, SE-901 87 Sweden

**Keywords:** Piperazine derivatives, Neurotoxicity, Mitochondrial dysfunction, Microtubules, P19 neurons

## Abstract

**Background:**

Piperazine derivatives such as BZP and TFMPP have been used as “party pill” substitutes for MDMA and are associated with neurological and cardiovascular toxicity. While their psychoactive effects are largely attributed to monoaminergic mechanisms, the cellular pathways underlying their toxicity are less well defined. Previous in vitro studies indicate mitochondrial dysfunction and oxidative stress, but potential effects on the neuronal cytoskeleton, specifically microtubules, have not been systematically investigated.

**Methods:**

The effects of MeOPP, BZP, pFPP and TFMPP were evaluated in retinoic acid–differentiated P19 mouse embryonal carcinoma–derived neurons using complementary assays of cell viability (calcein-AM), metabolic activity (MTT), membrane integrity (LDH), mitochondrial membrane potential (TMRE) and βIII-tubulin immunofluorescence. Key findings were evaluated in differentiated human SH-SY5Y neuroblastoma cells and in Caco-2 human colorectal adenocarcinoma cells. Tubulin polymerization was assessed in a complementary cell-free assay.

**Results:**

All compounds induced concentration-dependent toxicity, with marked differences in potency and efficacy. TFMPP was the most active compound across endpoints, producing early and sustained loss of mitochondrial membrane potential followed by reduced viability, cytoskeletal changes and increased membrane damage. BZP and pFPP showed moderate toxicity at higher concentrations, whereas MeOPP had limited effects. Time-course analysis demonstrated that mitochondrial depolarization preceded membrane damage. Reduced βIII-tubulin immunofluorescence in neuronal cells, together with inhibition of tubulin polymerization in a cell-free system, is consistent with effects on microtubule-related processes. Similar toxicity patterns were observed in SH-SY5Y cells, and cytotoxic effects of BZP and TFMPP were also detected in Caco-2 cells.

**Conclusions:**

Piperazine derivatives are associated with cellular toxicity characterized by early mitochondrial dysfunction and subsequent effects on the neuronal cytoskeleton. TFMPP showed the most consistent activity across models. The findings indicate that microtubule-related processes may contribute to toxicity and support further mechanistic studies beyond monoaminergic pathways.

**Supplementary Information:**

The online version contains supplementary material available at 10.1186/s40360-026-01183-3.

## Background

Novel psychoactive substances (NPS) continue to represent an important global public health challenge. New substances are detected each year, many with limited data on their pharmacology and toxicity [1, 2]. In addition, drugs sold on the recreational drug market may contain unknown or multiple active compounds, making clinical effects difficult to predict. This creates a need for experimental studies that clarify the toxic mechanisms of individual NPS and relevant drug combinations.

Among earlier stimulant-type NPS, piperazine derivatives including N-benzylpiperazine (BZP), 1-(3-trifluoromethylphenyl)piperazine (TFMPP), 1-(4-methoxyphenyl)piperazine (MeOPP) and related analogues as 1-(4-fluorophenyl)piperazine (pFPP) emerged as recreational drugs in the 2000s [3, 4]. They were commonly sold as “party pills” and marketed as legal alternatives or substitutes for MDMA [5].

Use of piperazine derivatives has been associated with acute neurological and cardiovascular toxicity. Reported effects include agitation, seizures, tachycardia, hypertension and, in some cases, severe or fatal intoxication [6, 7]. Toxicity may be further increased in cases of polydrug exposure, particularly when combined with other serotonergic substances [3, 8–10].

The pharmacological profiles of BZP and TFMPP are complementary but distinct. BZP exhibits amphetamine-like properties with monoamine-releasing effects [5], whereas TFMPP primarily acts through serotonergic mechanisms, including interaction with the serotonin transporter and activation of 5-HT receptors [11, 12]. Co-administration of BZP and TFMPP increases extracellular dopamine and serotonin in vivo in a pattern resembling that produced by MDMA [5], consistent with their historical use in combination in recreational “party pill” preparations. However, the mechanisms underlying piperazine-induced cellular toxicity remain less clearly defined. While their psychoactive effects are primarily linked to monoamine systems, available in vitro studies indicate that toxicity also involves intracellular pathways beyond neurotransmitter release and receptor activation. Mitochondrial dysfunction appears to be a central component of piperazine-induced toxicity. In non-neuronal and hepatic models, these compounds induce oxidative stress, mitochondrial impairment and apoptosis [13, 14], and related mechanisms have been implicated in neuronal cell models [15]. However, previous studies have mainly assessed responses at fixed exposure times and have rarely included cytoskeletal endpoints, leaving the mechanisms of piperazine-induced toxicity incompletely resolved.

Disruption of the neuronal cytoskeleton represents a critical but underexplored aspect of neurotoxicity. Neuronal viability and function depend on a dynamic microtubule network, in which βIII-tubulin is an important neuronal component. Structurally diverse small molecules, including certain psychoactive compounds, are known to alter microtubule dynamics [16, 17].

In this context, the related aromatic piperazine structures of BZP, TFMPP, MeOPP and pFPP raises the possibility of interactions with cytoskeletal targets, although this has not been systematically investigated.

To address these gaps, we investigated the in vitro toxicity of BZP, TFMPP, MeOPP and pFPP in retinoic acid (RA)-differentiated P19 mouse embryonal carcinoma (EC) cells (P19 neurons). P19 cells, originally isolated from a teratocarcinoma in C3H/He mice [18], differentiate into postmitotic neurons and glial-like cells upon retinoic acid (RA) treatment [19, 20] and provide a reproducible platform for mechanistic neurotoxicity studies in defined media [21, 22]. In our previous comparison of neuronal cell models, differentiated P19 cells developed a more extensive neuronal network and were more sensitive than RA-differentiated human SH-SY5Y neuroblastoma cells and nerve growth factor-treated rat PC12 pheochromocytoma cells for detecting several forms of chemical-induced neurotoxicity [21]. βIII-tubulin immunoreactivity has also been validated in P19-derived neurons as a fluorescence microplate readout of neurite-associated and cytoskeletal toxicity [23].

By combining multiple endpoints, the study characterized both overall cytotoxicity and specific toxic events, including mitochondrial dysfunction, membrane damage and neuronal cytoskeletal changes. Potential microtubule involvement was further assessed using a cell-free tubulin polymerization assay. Key endpoints were additionally assessed in differentiated SH-SY5Y cells and Caco-2 epithelial cells to determine whether the toxicity profile was reproducible in a human neuronal model and detectable in a human non-neuronal epithelial model.

## Material and methods

### Chemicals

MEM-α medium containing deoxyribonucleosides and ribonucleosides, MEM with Earle’s salts and L-glutamine, fetal bovine serum (FBS), penicillin–streptomycin, MEM non-essential amino acids (NEAA), Neurobasal medium, B27 supplement, N2 supplement, L-glutamine, Alexa Fluor 488-conjugated goat anti-rabbit IgG, calcein-acetoxymethyl ester (calcein-AM), and Hanks’ balanced salt solution (HBSS) with CaCl_2_ and MgCl_2_ were purchased from Invitrogen Life Technologies, Uppsala, Sweden. Minimum essential medium Eagle with Earle’s salts and sodium bicarbonate (EMEM), Dulbecco’s modified Eagle’s medium/Ham’s F12 nutrient mixture (1:1), all-trans retinoic acid, poly-D-lysine hydrobromide, and the piperazine derivatives 1-(4-methoxyphenyl)piperazine (MeOPP, purity ≥ 97%), N-benzylpiperazine (BZP, purity ≥ 97%), 1-(4-fluorophenyl)piperazine (pFPP, purity ≥ 98%), and 1-(3-trifluoromethylphenyl)piperazine (TFMPP, purity 95%), all used as free bases, as well as dimethyl sulfoxide (DMSO), thiazolyl blue tetrazolium bromide (MTT), and bovine serum albumin, were purchased from Sigma-Aldrich (Merck), Stockholm, Sweden. Rabbit polyclonal anti-βIII-tubulin antibodies were obtained from Covance, Princeton, NJ, USA. Formaldehyde solution (4%) in phosphate buffer was obtained from APL, Umeå, Sweden. The LDH Cytotoxicity Detection Kit was obtained from Roche Diagnostics, Mannheim, Germany, the TMRE Mitochondrial Membrane Potential Assay Kit from Abcam, Cambridge, UK, and the Tubulin Polymerization Assay Kit (Cat. #BK011P) from Cytoskeleton, Inc., Denver, CO, USA.

### Cell culture and neuronal differentiation

P19 mouse embryonal carcinoma cells, human neuroblastoma SH-SY5Y cells, and human colon adenocarcinoma Caco-2 cells were obtained from the European Collection of Cell Cultures (ECACC, Porton Down, UK). All cell lines were maintained at 37 °C in a humidified atmosphere with 5% CO_2_.

P19 cells (passages 14–28) were cultured in MEM-α medium containing deoxyribonucleosides and ribonucleosides, supplemented with 10% FBS, 100 U/mL penicillin, 100 µg/mL streptomycin, and 1% MEM non-essential amino acids (NEAA). The medium was replaced every 48 h, and cells were passaged every 96 h at 70–80% confluence. Neuronal differentiation was induced essentially as previously described [22, 24]. Briefly, cells were seeded at 1 × 10⁶ cells in bacterial-grade Petri dishes (Ø 92 mm; Sarstedt, Newton, NC, USA) in medium containing 5% FBS and 1 µM all-trans retinoic acid. The medium was replaced after 48 h. By day 4, cell aggregates had formed and were trypsinized and plated onto poly-D-lysine-coated (50 µg/mL) 96-well plates at a density of 16,000 cells/well in serum-free Neurobasal medium supplemented with 2% B27, 1 mM L-glutamine, 100 U/mL penicillin, and 100 µg/mL streptomycin. Half of the medium was replaced every 48 h, and cells were cultured for 7 days prior to experiments.

SH-SY5Y cells (passages 21–26) were maintained in EMEM supplemented with 10% FBS, 100 U/mL penicillin, 100 µg/mL streptomycin, 1% MEM NEAA, and 2 mM L-glutamine. Cells were passaged every 4–7 days and seeded at 10,000 cells/cm². For experiments, cells were plated in 96-well plates at 10,000 cells/well in 200 µL medium and allowed to attach for 24 h. The medium was then replaced with serum-free DMEM/F12 (1:1) containing N2 supplement and 1 µM retinoic acid to induce differentiation. Cells were cultured under these conditions for 72 h prior to compound exposure.

Caco-2 cells (passages 83–90) were cultured in MEM supplemented with 10% FBS, 100 U/mL penicillin, 100 µg/mL streptomycin, 1% MEM NEAA, and 2 mM L-glutamine. Prior to experiments, cells were seeded overnight in 96-well plates at a density of 1 × 10⁴ cells/well in medium containing 1% FBS.

### Preparation of the compounds

Stock solutions of MeOPP and BZP were prepared in Milli-Q water, whereas stock solutions of pFPP and TFMPP were prepared in DMSO. The final concentration of DMSO was maintained at 0.5% (v/v) in all relevant samples and vehicle controls. Immediately before the experiments, stock solutions were diluted in the appropriate culture medium for each cell line.

### Calcein-AM assay

Intracellular esterase activity was assessed using the fluorescence-based calcein-acetoxymethyl ester (calcein-AM) assay. Cells were washed once with PBS, followed by incubation with 1 µM calcein-AM in PBS for 1 h at room temperature. Fluorescence was measured using a FLUOstar Galaxy microplate reader (BMG Labtech) with excitation/emission at 485/520 nm.

### LDH release assay

Cell membrane integrity was assessed by measuring extracellular lactate dehydrogenase (LDH) activity using a cytotoxicity detection kit according to the manufacturer’s instructions. Briefly, 100 µL aliquots of culture medium were transferred to a new plate and mixed with 100 µL of reaction mixture. After incubation for 30 min at room temperature, absorbance was measured at 490 nm with a reference wavelength of 650 nm using a SPECTROstar Nano plate reader (BMG Labtech, Offenburg, Germany). Cells treated with 2% Triton X-100 for 30 min at 37 °C and 5% CO₂ were used as controls for maximal LDH release.

### MTT assay

Cell viability was assessed using the MTT reduction assay with minor modifications. Culture medium was replaced with 100 µL fresh medium, followed by addition of 10 µL MTT solution (5 mg/mL in PBS). After incubation for 3 h at 37 °C and 5% CO₂ protected from light, formazan crystals were solubilized by adding 100 µL of 0.01 M HCl in 10% SDS and incubating overnight at room temperature. Absorbance was measured at 570 nm using a SPECTROstar Nano plate reader.

### Mitochondrial membrane potential (ΔΨm) analysis

Changes in mitochondrial membrane potential were assessed using a TMRE (tetramethylrhodamine ethyl ester) assay kit. P19 neurons (2.4 × 10⁴ cells/well in 96-well plates) were treated with BZP, TFMPP, MeOPP, and pFPP for 24 h on day 7 in serum-free medium. FCCP (5 µM) was used as a positive control (10 min exposure). Cells were incubated with TMRE (500 nM) for 30–45 min at 37 °C and 5% CO₂, washed once with HBSS containing 0.2% bovine serum albumin, and fluorescence was measured using a FLUOstar Galaxy plate reader at 544/590 nm.

### βIII-tubulin immunofluorescence analysis

P19 neurons and differentiated SH-SY5Y cells were immunostained for the neuron-specific protein βIII-tubulin [25, 26], and fluorescence was quantified as previously described in a validated microplate-based assay for neurite- and cytoskeleton-associated toxicity in P19-derived neurons [23]. Cells were fixed in 4% formaldehyde for 30 min, washed with PBS, and permeabilized with 0.1% Triton X-100 in PBS for 5 min. After two washes in PBS, non-specific binding was blocked with 3% FBS in PBS for 30 min.

Primary antibodies against βIII-tubulin were diluted 1:500 in blocking solution and applied for 1 h, followed by three washes in PBS. Alexa Fluor 488-conjugated secondary antibodies were diluted 1:250 and applied for 1 h 20 min. Cells were washed three times in PBS and analysed. Control samples incubated with secondary antibody only were used to determine background fluorescence.

Total fluorescence intensity was measured using a FLUOstar Galaxy microplate reader (BMG Labtech, Offenburg, Germany) with excitation/emission at 485/520 nm. Representative fluorescence images were acquired using a Nikon Eclipse TE2000-U inverted microscope equipped with a Plan Fluor ELWD 20×/0.45 objective and a Nikon Digital Sight DS-5Mc camera (Tekno Optik AB, Skärholmen, Sweden). Cells treated with 2% Triton X-100 for 30 min at 37 °C and 5% CO₂ were used as controls for maximal cell death.

### Tubulin polymerization assay

Tubulin polymerization was assessed using a fluorescence-based assay according to the manufacturer’s instructions. Briefly, a black, flat-bottom, half-area 96-well plate (Corning Costar, Cat. 3686) was prewarmed at 37 °C for at least 10 min. Aliquots (5 µL) of test compounds at 10× working concentrations were added to the plate and incubated for 1 min at 37 °C. The final DMSO concentration was 0.1%.

Polymerization was initiated by adding 50 µL of ice-cold assay mixture containing 2 mg/mL porcine tubulin in 80 mM PIPES buffer (pH 6.9), 2 mM MgCl₂, 0.5 mM EGTA, 1 mM GTP, and 15% glycerol. Fluorescence was measured from the top using a FLUOstar Galaxy microplate reader (BMG Labtech, Offenburg, Germany) at 37 °C (excitation/emission 380/460 nm). Measurement settings included orbital shaking for 5 s prior to the first cycle (1 mm amplitude, 350 rpm), followed by 121 cycles with a cycle time of 60 s and three reads per well. Data were expressed as fluorescence units over time.

### Data and statistical analyses

Raw fluorescence and absorbance values were corrected for background by subtraction of assay-specific background signal. For plate-based assays, background was defined as signal from wells without cells or without detection reagent, whereas for immunofluorescence assays (βIII-tubulin), background was determined from wells incubated with secondary antibody only (no primary antibody). Data were subsequently normalized within each experiment to matched control or reference conditions, depending on the endpoint and analysis.

For viability-related endpoints (calcein fluorescence and βIII-tubulin immunofluorescence), values were normalized to the mean of matched untreated control wells and expressed as percentage of control (100%). Control values shown in figures represent independent wells not used for normalization.

For TMRE assays, different normalization approaches were used depending on experimental design. In concentration–response experiments, background-corrected fluorescence values were expressed as relative fluorescence units (RFU) following subtraction of signal from corresponding wells without TMRE. In time-course experiments, TMRE fluorescence was normalized to matched control at each time point and expressed as percentage of control.

For LDH assays, two normalization approaches were used. In direct LDH readouts, absorbance values were normalized to the mean signal from Triton X-100-treated wells and expressed as percentage of maximal LDH release (Triton X-100 = 100%). For comparative cytotoxicity analyses, LDH and MTT data were normalized to a common scale defined by untreated control (0%) and Triton X-100-induced maximal toxicity (100%).

In this case, LDH cytotoxicity (membrane damage) was calculated as:

$$\eqalign{ & \>(Ab{s_{sample}}\> - \>Ab{s_{control}})\>/\> \cr & \,\,\,\,(Ab{s_{Triton}}\> - \>Ab{s_{control}})\> \times \>\>100 \cr}$$

and MTT cytotoxicity (metabolic impairment) as:

$$\eqalign{ & \>(Ab{s_{control}}\> - \>Ab{s_{sample}})\>/\> \cr & \,\,\,\,\,(Ab{s_{control}}\> - \>Ab{s_{Triton}})\> \times \>\>100 \cr}$$

Tubulin polymerization was monitored as fluorescence intensity over time (0–120 min). Each condition was measured in duplicate and mean values were used for analysis. AUC was calculated from 10 to 120 min using the trapezoidal method using GraphPad Prism for macOS version 11 (GraphPad Software, San Diego, CA), without baseline subtraction. The first 10 min were excluded to allow stabilization of the fluorescence signal and completion of the initial lag phase before quantification. Thus, integration was restricted to the common growth and steady-state phases to allow comparable quantification of net tubulin polymerization. AUC values are expressed as fluorescence units × time (FU × min) and used as a quantitative measure of overall tubulin polymerization.

Concentration–response data were analysed in GraphPad Prism by nonlinear least-squares regression using three-parameter log(inhibitor) versus response models and, where appropriate, compared with four-parameter variable-slope models using the extra sum-of-squares F test. The simpler model was selected unless the more complex model provided a significantly better fit (*p* < 0.05). No weighting was applied, and replicate values were treated as individual data points.

Data are presented as mean ± SD from independent experiments, with *n* indicating the number of separate experiments, unless otherwise stated. Each experiment included 2–5 technical replicates, depending on assay and experimental design.

Statistical analyses were performed in GraphPad Prism. A two-sided *p* value < 0.05 was considered statistically significant. Single-factor experiments were analysed using ordinary one-way ANOVA followed by either Dunnett’s or Šídák’s multiple-comparisons test, depending on the type of comparisons. Dunnett’s test was used when all treatments were compared with a single common control. Šídák’s test was used for selected pairwise comparisons, including experiments in which compounds were compared with their corresponding untreated or vehicle controls. Time-course experiments were analysed using two-way repeated-measures ANOVA with treatment and time as factors, followed by Šídák’s multiple-comparisons test for comparisons between treatment groups at individual time points. Direct comparisons involving two matched groups were analysed using unpaired two-tailed Student’s t-tests.

## Results

### TFMPP shows the highest cytotoxic potency in P19 neurons

**Fig. 1:**
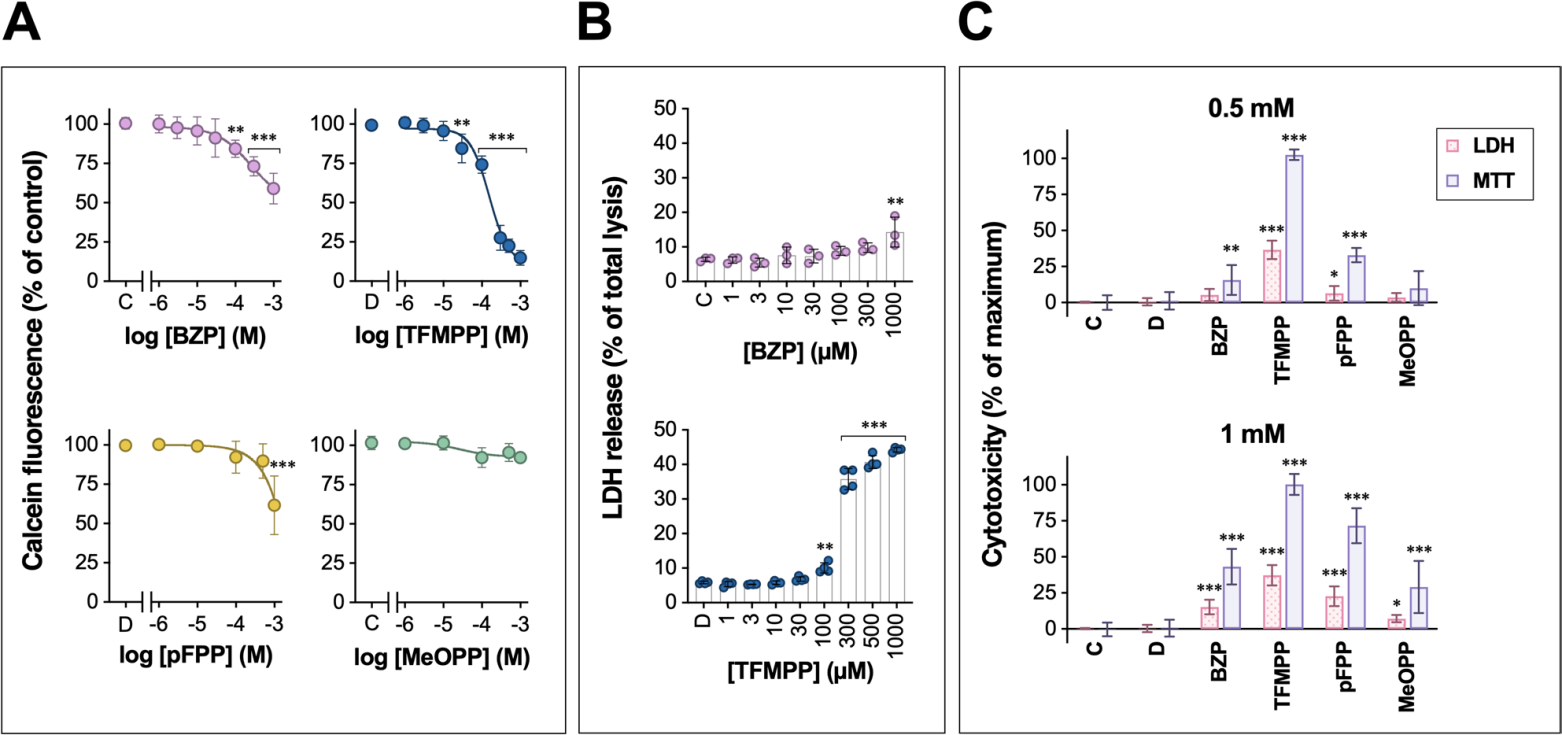
Piperazine effects on viability, membrane integrity and metabolic impairment in P19 neurons. (**A**) Cell viability assessed by calcein fluorescence after 24 h exposure to BZP, TFMPP, pFPP and MeOPP (1 µM–1 mM), expressed as percentage of matched control. TFMPP reduced viability at ≥ 0.03 mM, BZP at 0.1–1 mM, pFPP at 1 mM, and MeOPP at 0.1 and 1 mM. Concentration–response data in panel A were fitted by nonlinear regression. TFMPP was fitted using a variable slope model, whereas BZP, pFPP and MeOPP were fitted by a three-parameter model. (**B**) Membrane integrity assessed by LDH release after 24 h exposure, expressed as percentage of total lysis (Triton X-100 = 100%). TFMPP increased LDH release in a concentration-dependent manner, whereas BZP produced weaker effects. (**C**) Cytotoxicity assessed by LDH release and MTT reduction following exposure to 0.5 or 1 mM of the piperazine compounds. Values are expressed relative to maximal toxicity (untreated control = 0%, Triton X-100 = 100%). TFMPP produced the greatest cytotoxicity at both concentrations, whereas pFPP showed intermediate effects and BZP and MeOPP produced weaker responses. Data are presented as mean ± SD. Throughout the figure, BZP and MeOPP were compared with untreated control cells (C; medium only), whereas TFMPP and pFPP were compared with vehicle control cells (D; 0.5% DMSO). Statistical analyses were performed using ordinary one-way ANOVA followed by Šídák’s multiple-comparisons test. Significant differences from the corresponding control are indicated by asterisks (*p < 0.05, **p < 0.01, ***p < 0.001). The number of independent experiments varied between assays, with *n* = 4–6 for calcein fluorescence, *n* = 3–4 for LDH release, *n* = 8–9 for membrane damage, and *n* = 6–7 for metabolic impairment

To establish a baseline toxicity profile in P19-derived neurons, MeOPP, BZP, pFPP and TFMPP (structures shown in Additional file 1 (Figure S1)) were compared after 24 h exposure using three complementary endpoints: calcein-AM fluorescence (Fig. 1A), LDH release (Fig. 1B), and cytotoxicity based on membrane damage (LDH assay) and metabolic impairment (MTT assay) at 0.5 and 1 mM (Fig. 1C).

Calcein fluorescence revealed concentration-dependent reductions in viability for all compounds, with distinct potency and efficacy profiles. TFMPP was the most potent, reducing viability from 30 µM and at all higher concentrations, reaching ~ 15% of control at 1 mM. BZP produced significant effects from 100 µM, with viability reduced to ~ 59% at 1 mM, whereas pFPP affected viability only at 1 mM (~ 62% of control). MeOPP induced small but significant reductions at 100 µM and 1 mM, with limited maximal effect (~ 92–93% of control). Concentration–response data were fitted by nonlinear regression, with TFMPP described by a variable-slope model and BZP, pFPP and MeOPP by three-parameter models. Based on maximal effects in the calcein assay, the rank order of efficacy was TFMPP >>> BZP ≈ pFPP > MeOPP.

LDH release showed compound-dependent effects on membrane integrity. TFMPP did not significantly affect LDH release at 0.001–0.03 mM but increased LDH release from 0.1 mM and above, reaching ~ 44% of total lysis at 1 mM. In contrast, BZP had no effect up to 0.3 mM and produced a modest increase only at 1 mM (~ 14% of total lysis). Thus, TFMPP induced a markedly greater loss of membrane integrity than BZP.

To compare membrane damage (LDH) and metabolic impairment (MTT), cytotoxicity was expressed relative to maximal toxicity (untreated control = 0%, Triton X-100 = 100%). At 0.5 mM, TFMPP produced marked increases in both membrane damage and metabolic impairment, whereas pFPP induced moderate metabolic impairment with only limited membrane damage and BZP and MeOPP produced only minor responses.

For most compounds, cytotoxicity estimated from the MTT assay exceeded that estimated from LDH release, suggesting that metabolic impairment preceded extensive membrane damage. At 1 mM, cytotoxicity increased for all compounds, with TFMPP producing the largest effect, followed by pFPP, whereas BZP and MeOPP showed weaker responses. Thus, across endpoints, TFMPP consistently exhibited the highest cytotoxic efficacy, whereas MeOPP showed low efficacy despite measurable effects on viability.

### Piperazine toxicity profiles in differentiated SH-SY5Y cells show similarities to those in P19 neurons

**Fig. 2:**
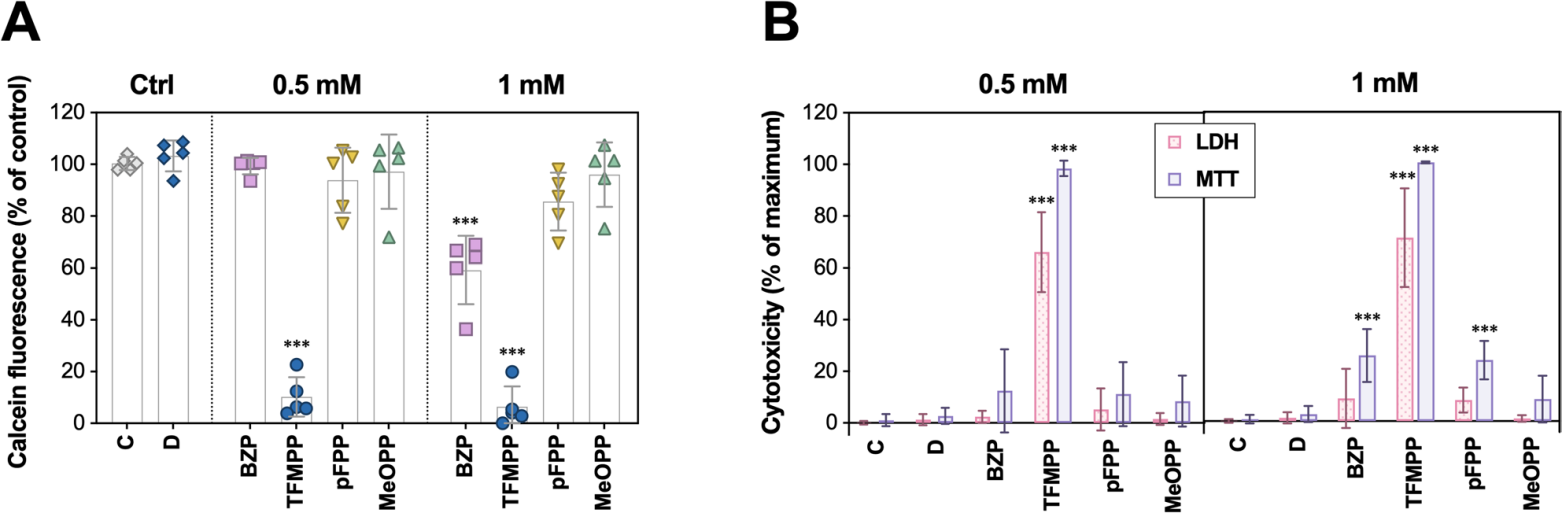
Piperazine effects on viability and cytotoxicity in differentiated SH-SY5Y cells. (**A**) Cell viability assessed by calcein fluorescence after 24 h exposure to BZP, TFMPP, pFPP and MeOPP at 0.5 and 1 mM, expressed as percentage of matched control. TFMPP markedly reduced viability at both concentrations, whereas BZP reduced viability at 1 mM only. (**B**) Cytotoxicity assessed by LDH release and MTT reduction following exposure to 0.5 or 1 mM of the piperazine compounds. Values are expressed relative to maximal toxicity (untreated control = 0%, Triton X-100 = 100%). TFMPP produced pronounced cytotoxic effects in both assays at both concentrations. BZP and pFPP produced greater effects in the MTT assay than in the LDH assay, indicating metabolic impairment in the absence of extensive membrane damage, whereas MeOPP showed only weak effects. Data are presented as mean ± SD (*n* = 5–8 independent experiments). Statistical analyses were performed using ordinary one-way ANOVA followed by Šídák’s multiple-comparisons test. Significant differences from the corresponding control are indicated by asterisks (*p < 0.05, **p < 0.01, ***p < 0.001). Throughout the figure, BZP and MeOPP were compared with the untreated control (C, medium only), whereas TFMPP and pFPP were compared with the vehicle control (D, 0.5% DMSO)

To assess whether the toxicity profiles observed in P19 neurons were conserved in a human neuronal model, we examined the effects of the piperazines in differentiated SH-SY5Y cells following 24 h exposure using the same cytotoxicity endpoints (Fig. 2).

Calcein fluorescence (Fig. 2A) showed a compound-dependent reduction in viability. TFMPP markedly reduced viability at both 0.5 and 1 mM, reaching near-complete loss of viable cells (~ 3–7% of control). BZP had no significant effect at 0.5 mM but reduced viability at 1 mM (~ 59% of control).

In contrast, pFPP and MeOPP did not significantly affect viability at either concentration. Based on maximal effects in the calcein assay, the rank order of efficacy was TFMPP >>> BZP > pFPP ≈ MeOPP.

Membrane integrity assessed by LDH release (Fig. 2B) showed a significant overall treatment effect at both concentrations. TFMPP caused pronounced membrane damage, reaching ~ 65–70% of maximal toxicity at both concentrations. In contrast, BZP, pFPP and MeOPP did not significantly increase LDH release at either concentration.

Metabolic impairment assessed by MTT reduction (Fig. 2B) also differed between treatments. At 0.5 mM, only TFMPP produced significant effects, reaching ~ 96% of maximal toxicity. At 1 mM, TFMPP remained near maximal toxicity (~ 97%), whereas BZP and pFPP produced smaller but significant effects (~ 25% and ~ 23%, respectively). MeOPP remained without significant effect.

Thus, TFMPP consistently induced both metabolic impairment and membrane damage in differentiated SH-SY5Y cells. In contrast, BZP and pFPP produced significant metabolic impairment at 1 mM without corresponding significant LDH release, indicating that metabolic dysfunction occurred in the absence of extensive membrane damage. MeOPP showed minimal effects across all endpoints.

### Piperazine toxicity extends to Caco-2 cells

**Fig. 3:**
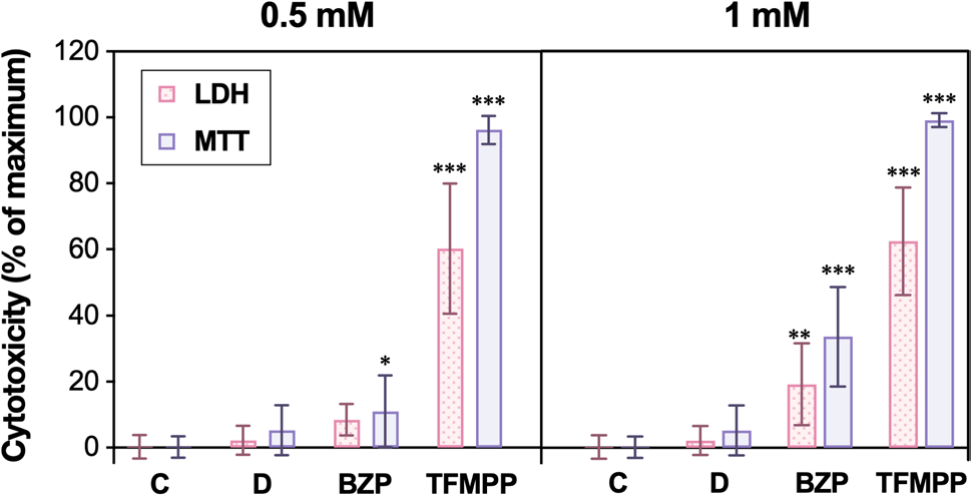
Cytotoxic effects of BZP and TFMPP in Caco-2 human colorectal adenocarcinoma cells. Cytotoxicity assessed by LDH release and MTT reduction following 24 h exposure to BZP or TFMPP at 0.5 and 1 mM. Values are expressed relative to maximal toxicity (untreated control = 0%, Triton X-100 = 100%). TFMPP produced pronounced cytotoxicity in both assays at both concentrations. BZP induced moderate but significant increases in both membrane damage and metabolic impairment, with greater effects at 1 mM. C denotes untreated control cells (medium only), whereas D denotes vehicle control cells exposed to 0.5% DMSO. Data are presented as mean ± SD (*n* = 9 independent experiments for LDH, and *n* = 7 for MTT). Statistical analyses were performed using ordinary one-way ANOVA followed by Šídák’s multiple-comparisons test. Significant differences from the corresponding control are indicated by asterisks (*p < 0.05, **p < 0.01, ***p < 0.001)

To assess whether the toxicity profiles observed in neuronal cells extended to a non-neuronal model relevant to intestinal exposure, BZP and TFMPP were evaluated in Caco-2 cells. Cytotoxicity was assessed by LDH release and MTT reduction following 24 h exposure to 0.5 and 1 mM of BZP or TFMPP (Fig. 3).

TFMPP induced marked toxicity at both concentrations, producing pronounced metabolic impairment and membrane damage at 0.5 and 1 mM. MTT reduction approached maximal values (~ 91–94%), whereas LDH release reached ~ 58–60% of maximal toxicity. BZP produced more moderate but significant effects in both assays, with greater responses at 1 mM than at 0.5 mM. MTT reduction increased from ~ 11% to ~ 34%, whereas LDH release increased from ~ 8% to ~ 19%. Overall, TFMPP consistently produced greater toxicity than BZP across both endpoints and concentrations, and both compounds produced more pronounced metabolic impairment than membrane damage.

### TFMPP induces early mitochondrial depolarization before detectable membrane damage in P19 neurons

**Fig. 4:**
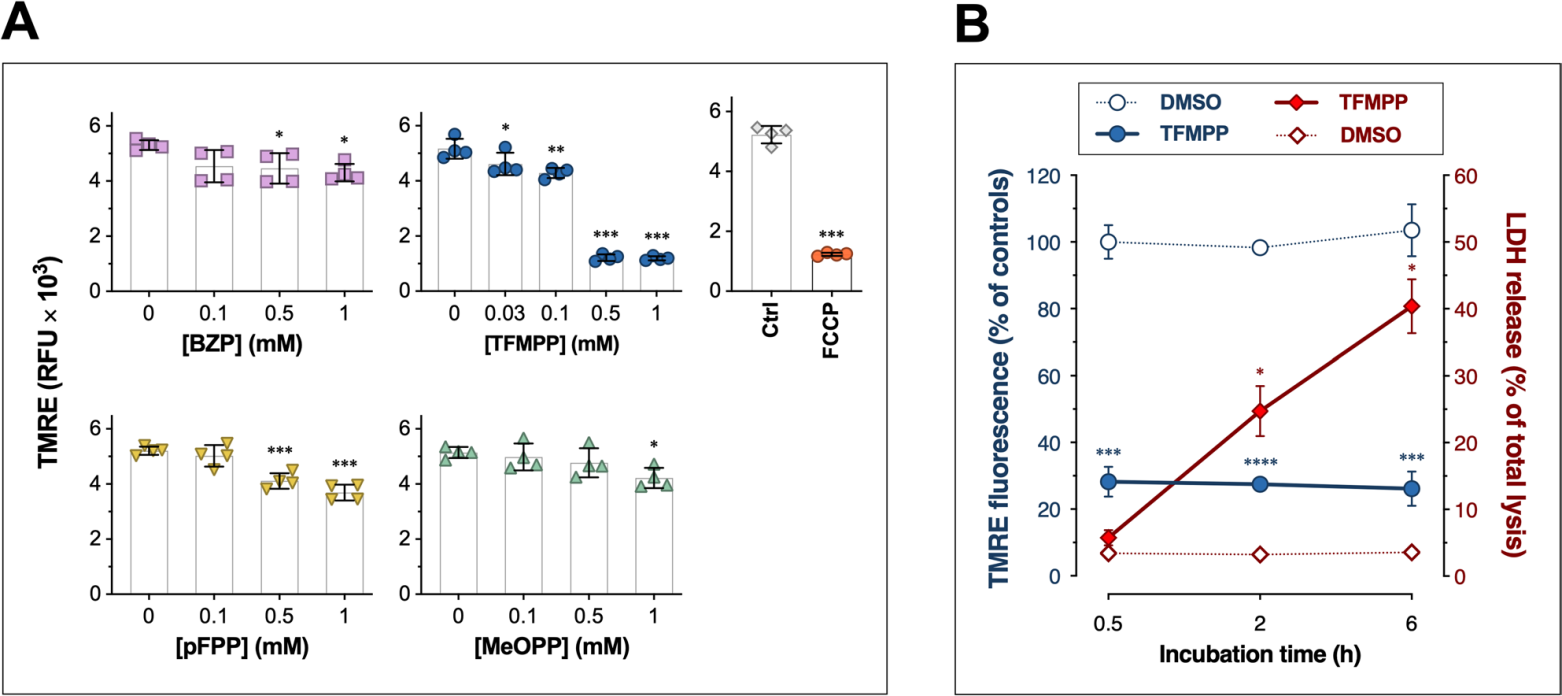
Piperazine effects on mitochondrial membrane potential in P19 neurons. (**A**) Mitochondrial membrane potential (ΔΨm) assessed by TMRE fluorescence following 24 h exposure to BZP, TFMPP, pFPP and MeOPP (0.03–1 mM for TFMPP; 0.1–1 mM for the other compounds), expressed as RFU × 10^3^. TFMPP induced a pronounced concentration-dependent reduction in TMRE fluorescence, whereas BZP and pFPP produced weaker effects and MeOPP showed minimal impact. FCCP (5 µM, 10 min) was used as a positive control. (**B**) Time-dependent effects of TFMPP (0.5 mM) on mitochondrial membrane potential (left y-axis, TMRE fluorescence; % of control) and membrane integrity (right y-axis, LDH release; % of total lysis) during 0.5–6 h exposure. TFMPP produced an early and sustained reduction in TMRE fluorescence, which had already decreased to approximately 28% of control at the earliest time point examined (0.5 h), whereas LDH release remained near basal levels initially and increased progressively over time. Data are presented as mean ± SD. Panel A was analysed using ordinary one-way ANOVA followed by Dunnett’s multiple-comparisons test and an unpaired two-tailed t-test for FCCP versus control. Panel B was analysed using two-way repeated-measures ANOVA (treatment × time) followed by Šídák’s multiple-comparisons test. Symbols indicate statistically significant differences from the corresponding controls (*p < 0.05, **p < 0.01, ***p < 0.001)

To determine whether mitochondrial dysfunction contributes to the observed cytotoxic effects, mitochondrial membrane potential (ΔΨm) was assessed using TMRE fluorescence in P19 neurons (Fig. 4).

The TMRE concentration-response and time-course experiments included four and three independent experiments, respectively.

At 24 h (Fig. 4A), the compounds differed markedly in their effects on TMRE fluorescence. TFMPP produced the strongest and most concentration-dependent reduction, with significant effects already at 0.03 mM and reductions at 0.5–1 mM (~ 4 × 10^3^ RFU below control) comparable to those produced by the mitochondrial uncoupler FCCP. BZP caused smaller but consistent reductions across the tested concentration range, reaching ~ 0.7–1.0 × 10^3^ RFU below control at 0.1–1 mM. pFPP showed intermediate effects, with significant reductions at 0.5 and 1 mM (~ 1.1 and ~ 1.5 × 10^3^ RFU below control, respectively). MeOPP showed only minor effects, reaching significance at 1 mM (~ 0.9 × 10^3^ RFU below control). Based on maximal effects, the rank order of efficacy for disruption of mitochondrial membrane potential was TFMPP >>> pFPP > BZP > MeOPP.

To assess temporal dynamics, TMRE fluorescence and LDH release were measured during exposure to TFMPP (0.5 mM) for 0.5–6 h (Fig. 4B). Prior to treatment, TMRE fluorescence was defined as 100% in both groups. At the earliest time point examined (0.5 h), TMRE fluorescence had already decreased to ~ 28% of matched control values and remained suppressed throughout the experiment. Consistent with this pattern, two-way repeated-measures ANOVA showed a significant effect of treatment, but no effect of time or treatment × time interaction, indicating a rapid and sustained loss of ΔΨm.

In contrast, LDH release remained close to basal levels at 0.5 h and increased progressively over time, reaching ~ 25% of total lysis at 2 h and ~ 40% at 6 h. Two-way repeated-measures ANOVA showed significant effects of treatment and time, together with a significant treatment × time interaction, indicating that membrane damage developed progressively during TFMPP exposure.

Together, these data show that TFMPP induces an early collapse of mitochondrial membrane potential followed by later membrane damage.

### Piperazines disrupt neuronal βIII-tubulin integrity and inhibit tubulin polymerization

**Fig. 5:**
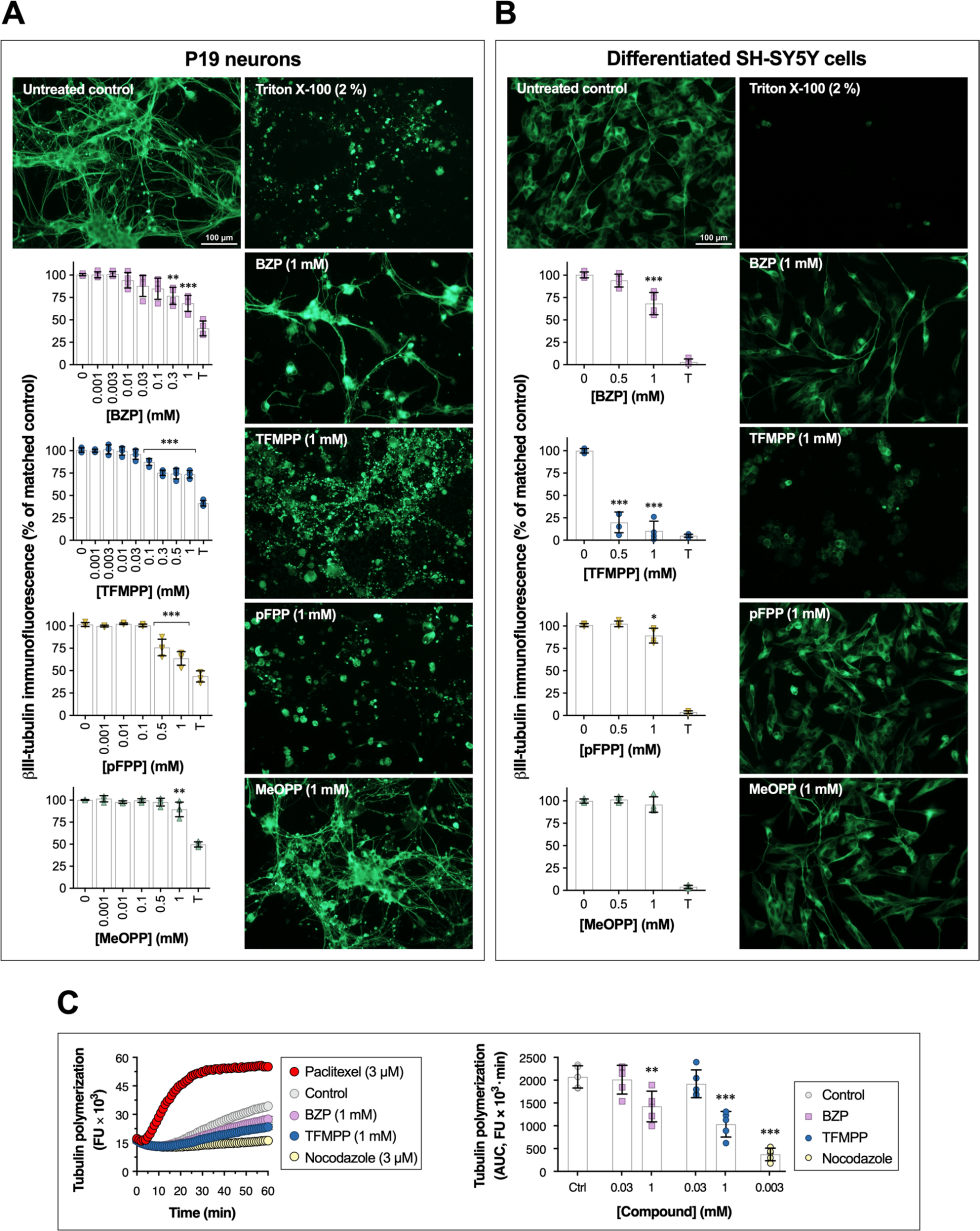
Piperazine effects on βIII-tubulin immunofluorescence and tubulin polymerization. (**A**) βIII-tubulin immunofluorescence in P19 neurons after 24 h exposure to BZP, TFMPP, pFPP and MeOPP. Representative fluorescence microscopy images (20× magnification; scale bars = 100 μm) are shown for untreated control, Triton X-100 (2%), and 1 mM of each compound, together with quantification of βIII-tubulin immunofluorescence. TFMPP significantly reduced βIII-tubulin immunofluorescence from 0.1 mM, BZP from 0.3 mM, pFPP from 0.5 mM, and MeOPP at 1 mM only. (**B**) βIII-tubulin immunofluorescence in differentiated SH-SY5Y cells after 24 h exposure to the same compounds. Representative images are shown for untreated control, Triton X-100 (2%) and 1 mM of each compound, together with quantification of βIII-tubulin immunofluorescence at 0.5 and 1 mM. TFMPP markedly reduced βIII-tubulin immunofluorescence at both concentrations, whereas BZP and pFPP showed smaller effects at 1 mM only and MeOPP showed no significant effect. (**C**) In vitro tubulin polymerization. Polymerization curves are shown for control, paclitaxel (3 µM; positive control), nocodazole (3 µM; negative control), BZP (1 mM) and TFMPP (1 mM). Polymerization was quantified as area under the curve (AUC) from 10 to 120 min. BZP and TFMPP did not significantly affect polymerization at 0.03 mM, but both reduced polymerization at 1 mM, with a stronger effect of TFMPP. Data are presented as mean ± SD. Statistical analyses were performed using ordinary one-way ANOVA followed by Dunnett’s multiple-comparisons test. Symbols indicate statistically significant differences from the corresponding controls (*p < 0.05, **p < 0.01, ***p < 0.001). Throughout the figure, BZP and MeOPP were compared with untreated control cells (medium only), whereas TFMPP and pFPP were compared with vehicle control cells (0.5% DMSO). T denotes Triton X-100 (2%)

To examine whether the piperazines affected neuronal cytoskeletal integrity, βIII-tubulin immunofluorescence was assessed after 24 h exposure in P19 neurons and differentiated SH-SY5Y cells, followed by a cell-free tubulin polymerization assay (Fig. 5).

In P19 neurons (Fig. 5A), all four compounds reduced βIII-tubulin immunofluorescence, but with clear differences in concentration dependence and efficacy. TFMPP produced significant reductions at concentrations ≥ 0.1 mM, with progressively larger effects at higher concentrations. At 1 mM, βIII-tubulin immunofluorescence was reduced by ~ 32% with BZP and ~ 27% with TFMPP. pFPP showed a steeper concentration dependence, with significant effects at 0.5 and 1 mM and the largest reduction at the highest concentration (~ 38% below control). MeOPP showed only minor effects, reaching significance at 1 mM (~ 11% below control). 

The number of independent experiments varied between assays, with *n* = 4–6 for βIII-tubulin immunofluorescence in P19 neurons, *n* = 4 for differentiated SH-SY5Y cells, and *n* = 5–6 for tubulin polymerization.

Representative images were consistent with these findings, showing reduced βIII-tubulin immunofluorescence and disruption of the neuronal network after piperazine exposure, whereas Triton X-100 caused marked loss of βIII-tubulin immunofluorescence.

In differentiated SH-SY5Y cells (Fig. 5B), the pattern was more selective. TFMPP produced a pronounced reduction in βIII-tubulin immunofluorescence at both 0.5 and 1 mM (~ 80% and ~ 90% below control, respectively). In contrast, BZP and pFPP showed smaller effects at 1 mM only (~ 32% and ~ 12% below control, respectively), whereas MeOPP did not significantly affect βIII-tubulin immunofluorescence at either concentration. Representative images similarly showed the strongest disruption after TFMPP exposure, while Triton X-100 again produced near-complete loss of signal. Thus, across the two neuronal models, TFMPP showed the most consistent effect, whereas BZP and pFPP produced weaker or more concentration-restricted changes and MeOPP remained least active.

To determine whether these cellular changes were associated with direct effects on tubulin assembly, tubulin polymerization was assessed in vitro (Fig. 5C). Representative polymerization curves recorded over 60 min showed the expected faster polymerization kinetics and higher plateau than control with paclitaxel, reaching ~ 55 × 10^3^ FU at the final time point versus ~ 34 × 10^3^ FU in control wells. In contrast, nocodazole markedly suppressed polymerization throughout the recording period and remained near ~ 16 × 10^3^ FU at 60 min. BZP and TFMPP at 1 mM produced slower polymerization kinetics and lower final signals than control, reaching ~ 28 × 10^3^ and ~ 23 × 10^3^ FU, respectively.

Quantification of overall tubulin polymerization as area under the curve (AUC) over a 10–120 min interval showed that neither BZP nor TFMPP affected polymerization at 0.03 mM. Under control conditions, AUC was ~ 2100 FU × 10^3^·min. At 1 mM, AUC was reduced to ~ 1450 FU × 10^3^·min with BZP, and ~ 1060 FU × 10^3^·min with TFMPP, corresponding to reductions of ~ 31% and ~ 49%, respectively. Nocodazole produced the strongest inhibition, reducing AUC to ~ 400 FU × 10^3^·min (~ 81% below control).

Together, these findings show that piperazines alter neuronal βIII-tubulin integrity in intact cells and that, at higher concentrations, BZP and TFMPP can inhibit tubulin polymerization in a cell-free system.

## Discussion

Piperazine derivatives remain toxicologically relevant recreational psychoactive substances, but their cellular mechanisms of toxicity are still incompletely resolved [27]. The present study shows that structurally related piperazine derivatives differ markedly in cellular toxicity despite sharing a common piperazine core and aromatic substitution.

TFMPP was the most toxic compound across the endpoints examined, producing the strongest loss of viability, membrane damage and mitochondrial depolarization in P19 neurons. Similar findings in differentiated SH-SY5Y cells and Caco-2 cells support that the observed toxicity was not restricted to a single cell model. Overall, BZP and pFPP showed intermediate effects, whereas MeOPP was comparatively weak under the present experimental conditions.

Interestingly, the overall toxicity ranking observed in the present study broadly parallels the relative lipophilicity of the compounds, with TFMPP representing the most lipophilic compound and MeOPP the least, whereas BZP and pFPP occupy an intermediate position. Thus, the greater toxicity of TFMPP and the comparatively weak effects of MeOPP are qualitatively consistent with these differences. Greater membrane partitioning and intracellular accumulation of more lipophilic compounds may contribute to differences in biological activity and toxicity. However, given the limited number of compounds examined and the likelihood that multiple mechanisms contribute to toxicity, this observation should be regarded as descriptive rather than evidence of a direct structure–toxicity relationship.

The rank order of toxicity observed here is consistent with previous in vitro studies of piperazine designer drugs. In H9c2 cardiomyoblasts, TFMPP was more potent than BZP, and MeOPP, with reported EC_50_ values of approximately 60 µM for TFMPP, 344 µM for BZP, and 570 µM for MeOPP after 24 h exposure [14]. Similar conclusions were reached in differentiated SH-SY5Y cells, where TFMPP was also identified as one of the most cytotoxic piperazines tested [15]. The higher potency of TFMPP is also consistent with functional neurotoxicity data from rat cortical cultures, where TFMPP inhibited neuronal network firing at substantially lower concentrations than BZP [28]. In P19 neurons, concentration–response analysis indicated inhibitory effects on both calcein fluorescence and βIII-tubulin immunofluorescence in the high micromolar range, consistent with previous in vitro studies of piperazine derivatives [13–15]. Since robust effects generally required high micromolar to millimolar concentrations, 0.5 and 1 mM were used in several follow-up experiments to allow comparison across endpoints and cell models under conditions with measurable toxicity, rather than to define precise potency differences.

A central mechanistic finding is that mitochondrial dysfunction occurred early in the toxic response. In P19 neurons, TFMPP caused a rapid and sustained decrease in TMRE fluorescence, whereas LDH release increased later. This temporal separation suggests that loss of mitochondrial membrane potential is not simply a consequence of terminal membrane disruption, but an early event in the progression of TFMPP-induced toxicity. Earlier piperazine studies have shown mitochondrial impairment, ATP depletion, calcium dysregulation and apoptotic signalling in cardiac and neuronal models [14, 15], and oxidative stress, mitochondrial impairment and apoptosis in hepatic and glial models [13, 29, 30]. However, these studies mainly assessed responses after fixed exposure periods, whereas the present data help resolve the temporal relationship between mitochondrial depolarization and later membrane damage. This supports a model in which early mitochondrial depolarization contributes to later metabolic impairment and membrane damage, rather than representing only a late marker of nonspecific cytotoxicity.

The most novel aspect of the study is evidence that piperazines affect neuronal cytoskeletal integrity and tubulin assembly. In both P19 neurons and differentiated SH-SY5Y cells, several piperazines reduced βIII-tubulin immunofluorescence, consistent with disruption of the neuronal cytoskeleton. This effect was most consistent for TFMPP across the two neuronal models but was also observed for BZP and pFPP. Importantly, the cell-based βIII-tubulin analysis was complemented by a cell-free tubulin polymerization assay showing that BZP and TFMPP inhibited tubulin polymerization at 1 mM. Together, these findings point to involvement of the microtubule system, although the two readouts reflect distinct experimental contexts.

These findings are biologically and chemically plausible. Microtubules are established targets for structurally diverse small molecules, and several pharmacologically relevant binding sites on tubulin, including the colchicine, taxane and vinca sites, have been described [17, 31, 32]. In addition, piperazine-containing aromatic compounds have been developed as tubulin polymerization inhibitors or colchicine-site agents, indicating that piperazine-containing scaffolds are present among compounds that target microtubules [33, 34]. Recent work on substituted phenethylamine designer compounds also suggests that some psychoactive compounds may interact with tubulin or affect microtubule polymerization, although these compounds are chemically distinct from the piperazines examined here [16].

However, the connection between reduced βIII-tubulin immunofluorescence in intact cells and inhibition of tubulin polymerization in a cell-free system is not clear.

At higher concentrations, reduced βIII-tubulin signal may partly reflect loss of neuronal cells or secondary structural collapse during cytotoxicity, rather than selective disruption of microtubules. Conversely, the polymerization assay measures inhibition of tubulin assembly under defined biochemical conditions and does not directly assess microtubule disassembly or degradation in cells. The present data therefore do not demonstrate that tubulin is a primary molecular target of BZP or TFMPP in intact cells.

Previous studies have shown that TFMPP and BZP induce oxidative stress, mitochondrial dysfunction and apoptotic signalling in several non-neuronal cell systems [13, 14, 29]. Reactive oxygen species may therefore provide an indirect link between mitochondrial dysfunction and the cytoskeletal alterations observed in the present study [35]. Oxidative modifications of tubulin have been shown to affect GTP binding and reduce microtubule polymerization, and oxidative stress resulting from mitochondrial dysfunction has been implicated in cytoskeletal disorganization [36]. Thus, mitochondrial dysfunction and associated oxidative stress could contribute indirectly to the reduced βIII-tubulin immunofluorescence and inhibition of tubulin polymerization observed here. However, since intracellular ATP levels, ROS formation and tubulin oxidation were not directly measured, the present data do not allow conclusions regarding whether these microtubule-related changes were secondary to cellular stress responses or reflected more direct effects on tubulin-associated processes.

Nevertheless, the combined findings suggest that microtubule dynamics may be affected under cytotoxic conditions. One possibility is that inhibition of tubulin assembly reflects interaction with functionally relevant binding sites on β-tubulin, which could, in a cellular context, destabilize microtubule turnover and contribute to structural collapse. This interpretation remains speculative and requires direct binding studies and more detailed analysis of microtubule structure, organization and integrity in cells treated with piperazine derivatives. Despite these uncertainties, the findings indicate that microtubule-related effects may contribute to piperazine-induced toxicity.

Consistent toxicity profiles across P19 neurons, differentiated SH-SY5Y cells and Caco-2 cells indicate that the toxicity of BZP and TFMPP was not confined to a single cell model. The Caco-2 data are relevant because these compounds are typically taken orally and Caco-2 cells are widely used as an intestinal epithelial model for drug absorption and barrier-related studies [37, 38]. However, the neuronal models were more informative for resolving mitochondrial and cytoskeletal endpoints, particularly in P19 neurons, which have previously been used for mechanistic neurotoxicity studies and for βIII-tubulin-based assessment of neurite-associated toxicity [23].

More broadly, NPS studies indicate that neuronal responses to psychoactive substances cannot always be explained by transporter pharmacology alone, but may involve additional functional and toxicological endpoints [39]. The present findings are consistent with this view and support the use of P19 neurons for studying intracellular toxicity mechanisms beyond monoamine release, transporter interactions and 5-HT receptor activity.

BZP and TFMPP have been used as “party pill” substitutes or alternatives to MDMA, and combined administration produces MDMA-like monoaminergic effects in vivo [5, 40]. However, our previous work in the same P19 neuronal model showed that MDMA-induced cytotoxicity occurred without detectable disruption of mitochondrial membrane potential under the conditions tested [41], whereas TFMPP produced early and sustained mitochondrial depolarization in the present study. These observations suggest that, although MDMA and piperazine derivatives can both be cytotoxic in P19 neurons, the intracellular sequence of toxic events may differ.

Several limitations should be acknowledged. First, most mechanistic experiments were performed at high micromolar to millimolar concentrations that are unlikely to be achieved under typical human exposure conditions. These concentrations were selected to permit comparison across endpoints and cell models and to identify potential mechanisms of toxicity, rather than to provide a quantitative model of in vivo exposure. Second, the assays used here reflect different aspects of cellular injury and do not distinguish between primary and secondary toxic events. Finally, although the present findings support involvement of microtubule-related processes, further studies are required to clarify the underlying mechanisms.

## Conclusions

In conclusion, the present study shows that piperazine-induced toxicity involves both early mitochondrial dysfunction and effects on neuronal microtubule-associated structure. TFMPP was consistently the most active compound across models, including early mitochondrial depolarization preceding detectable membrane damage in P19 neurons. In addition, reduced βIII-tubulin immunofluorescence and inhibition of tubulin polymerization at high concentrations indicate that microtubule-related effects may contribute under cytotoxic conditions. These findings support further evaluation of piperazine derivatives, with future studies needed to determine whether mitochondrial and cytoskeletal effects are mechanistically linked or occur in parallel.

## Supplementary Information

Below is the link to the electronic supplementary material.

Supplementary Material 1: Additional file 1. Figure S1. Chemical structures of the piperazine derivatives used in this study. Chemical structures of N-benzylpiperazine (BZP), 1-(3-trifluoromethylphenyl)piperazine (TFMPP), 1-(4-fluorophenyl)piperazine (pFPP) and 1-(4-methoxyphenyl)piperazine (MeOPP), illustrating variation in aryl substitution of the piperazine scaffold.

## Data Availability

All data supporting the findings of this study are included within the article and its supplementary information files.

## Abbreviations

BZP: N-benzylpiperazine
TFMPP: 1-(3-trifluoromethylphenyl)piperazine
MeOPP: 1-(4-methoxyphenyl)piperazine
pFPP: 1-(4-fluorophenyl)piperazine
NPS: Novel psychoactive substances
MDMA: 3, 4-methylenedioxymethamphetamine
RA: Retinoic acid
EC: Embryonal carcinoma
LDH: Lactate dehydrogenase
MTT: Thiazolyl blue tetrazolium bromide
TMRE: Tetramethylrhodamine ethyl ester
ΔΨm: Mitochondrial membrane potential
FCCP: Carbonyl cyanide-p-trifluoromethoxyphenylhydrazone
AUC: Area under the curve
RFU: Relative fluorescence units
FU: Fluorescence units
CI: Confidence interval
