# Supplementary material for: In vitro toxicity of piperazine derivatives involves mitochondrial dysfunction and microtubule-related changes in neuronal cell models
Source: BMC Pharmacol Toxicol. 2026 Jul 15;27:99. doi: 10.1186/s40360-026-01183-3 (PMC13377971; doi:10.1186/s40360-026-01183-3)
Supplement: Supplementary file 1 — Supplementary Material 1: Additional file 1. Figure S1. Chemical structures of the piperazine derivatives used in this study. Chemical structures of N-benzylpiperazine (BZP), 1-(3-trifluoromethylphenyl)piperazine (TFMPP), 1-(4-fluorophenyl)piperazine (pFPP) and 1-(4-methoxyphenyl)piperazine (MeOPP), illustrating variation in aryl substitution of the piperazine scaffold. [file 40360_2026_1183_MOESM1_ESM.pdf]

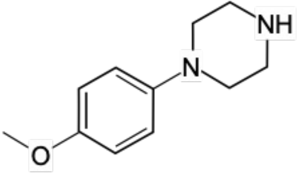

1-(4-Methoxyphenyl)piperazine
(MeOPP)

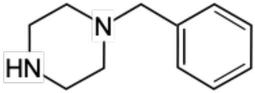

N-Benzylpiperazine
(BZP)

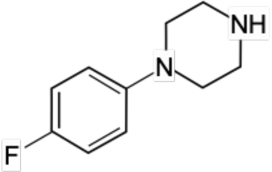

1-(4-Fluorophenyl)piperazine
(pFPP)

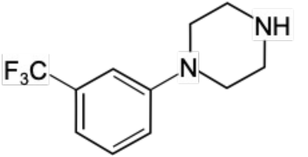

1-(3-Trifluoromethylphenyl)piperazine
(TFMPP)
